# Long-Term Changes in Nutrients and Mussel Stocks Are Related to Numbers of Breeding Eiders *Somateria mollissima* at a Large Baltic Colony

**DOI:** 10.1371/journal.pone.0095851

**Published:** 2014-04-29

**Authors:** Karsten Laursen, Anders Pape Møller

**Affiliations:** 1 Institute of Bioscience, Aarhus University, Rønde, Denmark; 2 Laboratoire d'Ecologie, Systématique et Evolution, CNRS UMR 8079, Université Paris-Sud, Orsay, France; CNRS, Université de Bourgogne, France

## Abstract

**Background:**

The Baltic/Wadden Sea eider *Somateria mollissima* flyway population is decreasing, and this trend is also reflected in the large eider colony at Christiansø situated in the Baltic Sea. This colony showed a 15-fold increase from 1925 until the mid-1990's, followed by a rapid decline in recent years, although the causes of this trend remain unknown. Most birds from the colony winter in the Wadden Sea, from which environmental data and information on the size of the main diet, the mussel *Mytilus edulis* stock exists. We hypothesised that changes in nutrients and water temperature in the Wadden Sea had an effect on the ecosystem affecting the size of mussel stocks, the principal food item for eiders, thereby influencing the number of breeding eider in the Christiansø colony.

**Methodology/Principal Finding:**

A positive relationship between the amount of fertilizer used by farmers and the concentration of phosphorus in the Wadden Sea (with a time lag of one year) allowed analysis of the predictions concerning effects of nutrients for the period 1925–2010. There was (1) increasing amounts of fertilizer used in agriculture and this increased the amount of nutrients in the marine environment thereby increasing the mussel stocks in the Wadden Sea. (2) The number of eiders at Christiansø increased when the amount of fertilizer increased. Finally (3) the number of eiders in the colony at Christiansø increased with the amount of mussel stocks in the Wadden Sea.

**Conclusions/Significance:**

The trend in the number of eiders at Christiansø is representative for the entire flyway population, and since nutrient reduction in the marine environment occurs in most parts of Northwest Europe, we hypothesize that this environmental candidate parameter is involved in the overall regulation of the Baltic/Wadden Sea eider population during recent decades.

## Introduction

Leakage of nutrients from farmland to the marine environment has changed the benthic community by increasing primary and secondary production. For example, primary production in Kattegat doubled between the 1950's and the early 1990's, and the biomass of fish increased six-fold during the same period and eight-fold in the Baltic Sea during 1900–1970 [1], [2]. However, the increase in nutrient load also had some undesirable effects in the marine ecosystem. Countries across Western Europe subsequently initiated programs in the 1970's and 1980's to reduce nutrient leakage from farmland, which led to reduced concentrations of nitrogen and phosphorus in the environment. In major rivers such as Elbe and the Rhine that receive water from large parts of European farmland and cities reduced concentrations of nutrients have been recorded since the late 1980's [3]–[5]. Continuous reductions in nutrients have also occurred in the North Sea and parts of the Baltic [6], [7]. Reduced nutrient loads affect the marine ecosystem by changing community structure of phytoplankton and benthos, although it has been difficult to predict the consequences for the ecosystem due to high annual variation [8]. However, studies of single waterbird species show clear relationships between nutrients and reproduction, recruitment, survival and breeding numbers [9].

Here we focus on the factors determining the size of the Baltic/Wadden Sea flyway population of eider *Somateria mollissima* that has decreased during the last two decades [10]. This decline was first reported from the wintering grounds and later from breeding areas [11], [12]. The reasons for the decline are poorly understood and epidemic outbreaks, parasite infestations, mass starvation, increased predation, and reduced survival of ducklings and adult females have all been suggested to be partly responsible for the decline [13]–[18]. However, no single study has so far shown that these factors account for the population decline, and a recent review [12] concluded that knowledge of the causes of declining survival of adult females is necessary for understanding the population decline.

**Figure 1:**
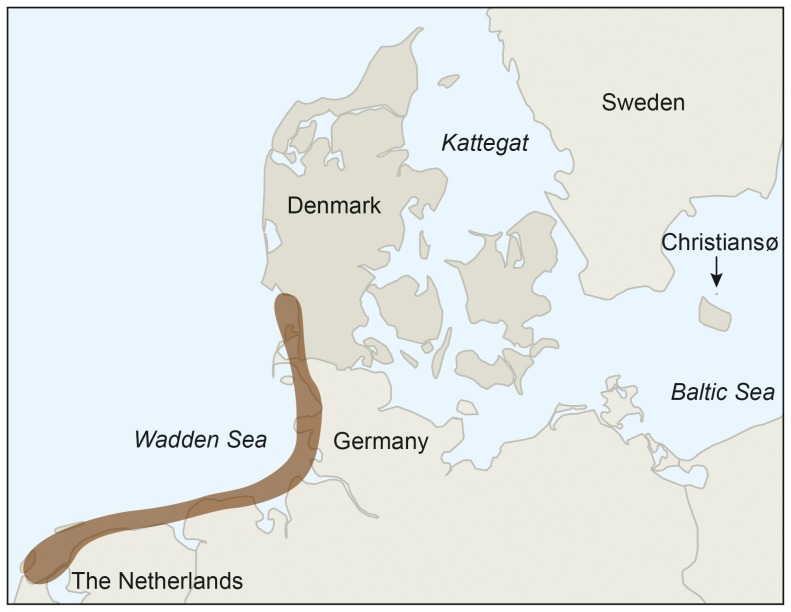
Eiders breeding at Christiansø are wintering in the Wadden Sea (toned brown) and in Kattegat. The eider colony at Christiansø in the Southern Baltic Sea was one of the largest colonies in the Baltic/Wadden Sea flyway population during the mid-1990's.

**Figure 2:**
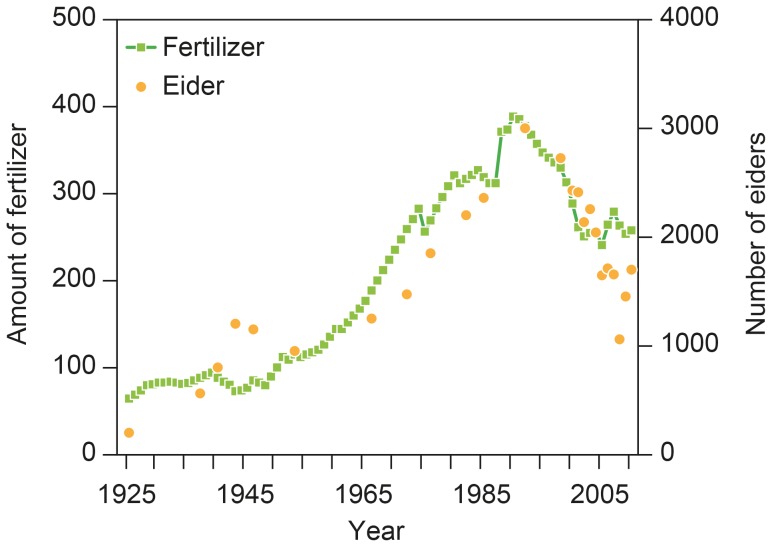
The number of breeding eiders at Christiansø and the amount of fertilizer used by Danish farmers (indexed as 1950 = 100) during 1925–2010.

The number of breeding eiders at Christiansø in the southern Baltic Sea (Fig. 1) is typical for several other colonies in the Baltic/Wadden Sea flyway population by showing a steady increase during the 1930's–1980's with a peak in the mid 1990's, followed by rapidly declining numbers (Fig. 2)[12], [19]. In addition eiders from this colony winter in the Danish waters (Kattegat and the Wadden Sea) as the vast majority of the flyway population [20]. The Christiansø colony has been monitored for 85 years and it is among the largest in the flyway breeding range counting up to 3,000 nesting females. The colony does not suffer from mammalian predation, and avian predation is limited due to close proximity to people. Thus female numbers returning to the colony may reflect conditions during the non-breeding season, i.e. ecological conditions at the wintering grounds. Female survival in the colony has not been estimated directly, but the annual number of breeders is supposed to reflect survival, since site fidelity of adult females is known to be almost 100% [21].

Eiders are capital breeders that build up most of their body reserves during winter for subsequent breeding [22]. When females arrive during spring they enter colonies and produce a clutch of 4–5 eggs that are incubated for about 26 days without the females leaving the nest to feed. Thus females must build up sufficient body reserves at the wintering grounds for breeding. Studies in the Wadden Sea show that wintering numbers of eiders are positively related to mussel stocks, and individuals feeding on mussels have better body condition than those taking other types of prey, which makes it attractive for eiders to feed on mussel beds [23]–[25]. Blue mussel *Mytilus edulis* can affect feeding conditions of eiders in at least two ways: (1) by increasing the flesh content and (2) by increasing the size of mussel stocks. First, the flesh content and reproduction in mussels are influenced by winter climate. During cold winters flesh content is large due to reduced respiration offering better foraging conditions for eiders than during mild winters [26]–[28]. Second, an increase in mussel stocks are based on large spatfall (production of eggs) in mussels, which are often initiated by cold winter climate making it possible for mussels to maintain large flesh content and produce large spatfall. Predation pressure is low in cold water and survival of mussel larvae during settling is high, giving rise to large cohorts 1–3 years after a cold winter. Thus cold winters maintain high flesh content in mussels that facilitate feeding conditions for eiders and increase their body condition for the subsequent breeding season. Cold winters increase mussel stock growth during the next 1–3 years and increase eider body conditions during the following years. However, flesh content and mussel growth depend on light, climatic conditions (temperature) and amount of nutrients that stimulate phytoplankton growth, the food of blue mussels [5], [29], [30]. Thus nutrients together with temperature are central variables in mussel stock growth and eider feeding conditions.

The objectives of this study were to test four predictions concerning the effects of changes in nutrient availability as indexed by fertilizer use in agricultural areas together with climate conditions have affected the number of eiders breeding at Christiansø through an increase in mussel stocks. First, we tested whether the concentration of nitrogen and phosphorus in the Wadden Sea was explained by the amount of fertilizer used on farmland and winter precipitation. Second, we tested if the amount of fertilizer together with climate (water temperature) could explain the size of mussel stocks in the Wadden Sea. Third, we tested if the number of breeding eiders at Christiansø could be predicted by linear effects of fertilizer used by farmers together with an effect of climate (water temperature). Fourth, we tested if mussel stocks in the Wadden Sea could explain the number of breeding eiders at Christiansø. We conducted these analyses by relying on extensive sampling in the Danish waters by the national Danish environmental monitoring programme NOVANA and the TMAP-programme in the Wadden Sea [5], [6].

The study was performed in the Baltic Sea at Christiansø, three rocky islands with a total area of 0.39 km^2^, of which two are inhabited (Fig. 1). The islands are covered by grass, bushes and a few trees. The vast majority of eiders breed on the inhabited islands near buildings, in gardens and along paths. Pets such as dogs and cats are not allowed on the islands, making it possible for eiders to breed in close vicinity to people. This reduces predation by gulls during incubation. A detailed description is given by Franzmann [21] and Lyngs [31]. The colony is normally not influenced by ice cover in the Baltic Sea, which affects clutch size and breeding success of eiders in the north-eastern part of the Baltic Sea [32] and at other sites with ice cover [33].

## Methods

### Ethics Statement

Eider nests, eggs and chicks are protected in Denmark according to the Wildlife Management and Hunting Act from 1931. We use existing data for this study that were collected by local wardens and ornithologists, who had permission to count birds according to the national monitoring program. The eiders at Christiansø are breeding in gardens and close to public paths and are used to people close to the nests. Counts of nests are easily made without disturbance of the birds, and counts of eggs in nests are performed by carefully lifting the females off the nests.

### Material

The number of eider nests was recorded since 1925 with annual intervals of 5.9 years (±0.4 SE) up to 1999 and thereafter annually until 2010. The number of breeding females was analysed in relation to the amount of fertilizer, mussel stocks and climate. In the analyses we included winter water temperature (mean °C for January–February) in the Wadden Sea measured in Marsdiep in the Dutch Wadden Sea [34], and updates. We used temperature measured in the Dutch Wadden Sea due to a long time series and because water temperature from this station reflects water temperature in the Danish waters and the western part of the Baltic Sea [35]. Information on precipitation during September-May was obtained from the Danish Meteorological Institute.

Information on nutrients as total nitrogen concentration (µg/l) was available from 1989 for the Danish waters, but Møller et al. [9] showed a close relationship between fertilizer (sum of artificial and natural fertilizers) use by Danish farmers and the concentration of nutrients in the Danish marine environment due to leakage of fertilizer from arable land to rivers and lakes and eventually the ocean. Information on fertilizer use by Danish farmers dates back to 1900 [36], and we use this as an indirect estimate of the amount of nutrients, which makes it possible to analyse the effects of nutrients for ecological variables for long time series. The annual amount of fertilizer use is estimated from autumn to spring the following year, e.g. the amount of fertilizer used in 1986 covers the amount used in autumn 1986 until spring 1987. Here the amount of fertilizer used in 1986/1987 is presented as 1986. From the Danish part of the Wadden Sea mussel stock data (metric tonnes) were available for 1987–2007 [25], [37], and nitrogen and phosphorus concentration for 1989–2009 (data from the Danish Ministry of the Environment).

### Statistical Procedures

We analyzed variation in and correlations among predictor variables to test for collinearity. We subsequently calculated variance inflation factors to determine whether collinearity posed a problem for the analyses. Furthermore, we tested for temporal trends in variables, and in case there was a significant temporal trend, we included year as a continuous predictor.

We made four major series of linear models. First, we tested whether N and P in the Danish Wadden Sea could be predicted by the amount of fertilizer used in agriculture by Danish farmers in year (i), year (i-1) and year (i-2). Time lags are used since fertilizer has to pass the soil to streams and lakes before it reaches coastal marine waters. These models all had predictors with variance inflation factors <5 [38].

Second, we tested if the amount of mussel stock in the Danish Wadden Sea could be predicted by the amount of fertilizer in year (i), year (i-1) and year (i-2), winter water temperature in year (i), year (i-1) and year (i-2) and the amount of mussel stock in year (i-1). We reduced these models by removing predictors relative to their associated P-values until all P-values were <0.10. These models all had predictors with variance inflation factors <10 and the final model <5 [38].

Third, we tested whether the abundance of breeding eiders at Christiansø could be predicted by the amount of fertilizer, winter water temperature and year. We included the amount of fertilizer in year (i), year (i-4) and (i-5) to account for effects of fertilizer on number of breeding females in the current year (i) and in year (i-4) and (i-5), to account for effects of fertilizer in the year of hatching for currently recruited females (we assume it takes 0–1 year for fertilizer to reach coastal waters and to stimulate mussel growth, and 3–4 years for eider ducklings to be mature [39]). Likewise, we included water temperature in year (i), year (i-4) and (i-5) to account for effects of temperature on number of breeding females in the current year (i) and effects of temperature in the year when recruiting females were hatched, i.e. year (i-4) and year (i-5). Finally, we included year as a continuous variable to account for temporal trends in variables other than fertilizer or temperature. We could only include variables in the tests that reflect these time lags in order to maintain variance inflation factors <10 and <5 in the final model [38].

Fourth, we tested if the number of breeding eiders in year (i) was predicted by the amount of mussel stock in year (i), year (i-1) and year (i-5). For justification for these time lags, see above. We only included variables that kept variance inflation factors <10 [38]. All analyses were made with JMP [40].

## Results

### Temporal Trends in Variables

The amount of fertilizer used by farmers increased from an index value of 64 in 1925 to about 100 in the late 1930's. In the early 1940's during the Second World War the use of fertilizer stabilized and later decreased, but in the 1950's it increased strongly again to reach a maximum index value of 377 in 1992, followed by a decrease to 250 in 2002 and later the amount stabilized until 2010 (Fig. 2). In the Wadden Sea the concentration of total nitrogen in spring increased during 1989–2000 from 755 µg/l to 2,141 µg/l after which it decreased to 1,297 µg/l in 2008. During the same period total phosphorus decreased from 55 µg/l in 1989 to 10–20 µg/l during 1990 to 2002 and <10 µg/l in 2010. Mussel stocks in the Wadden Sea increased from 40,000 metric tonnes in 1986 to 117,000 metric tonnes in 1994, while varying considerably during the following years, and it decreased to finally reach 12,000 metric tonnes in 2007. The breeding population of eiders at Christiansø increased from 200 females in 1925 to about 1,200 females in 1943. During the following years numbers declined, but increased to 3,000 females in 1992 followed by a decline to 1,060 females in 2008, and a small recent recovery (Fig. 2). All data are given in Supporting data (Table S1)

### Summary Statistics, Correlations and Temporal Tends in Data

All predictor variables showed considerable variation during the study period (Table S2). There were strong correlations among several variables (Table S3) preventing their inclusion as predictors in the same analyses. All variables with the exception of water temperature showed significant temporal trends (Table S4).

### Fertilizer Use on Farmland and N and P in the Marine Environment

**Figure 3:**
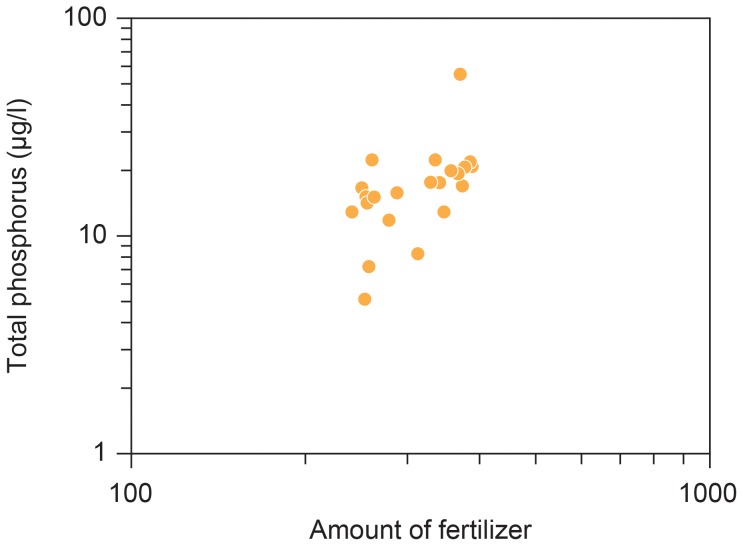
Total concentration of phosphate (µg/l) in the Danish Wadden Sea in year (i) in relation to fertilizer use in agriculture (1950 = 100) in the previous year (year (i-1)).

Only concentration of total P during spring in the marine environment was predicted by fertilizer and only in the previous year (Fig. 3; *F* = 9.26, d.f. = 1, 18, *r* ^2^ = 0.30, *P* = 0.007, estimate (SE) = 0.0018 (0.0006)), while there was no similar effect for concentration of total N (*F* = 0.04, d.f. = 1, 18, *r* ^2^ = −0.05, *P* = 0.85) or for the different time lags (results not shown). This makes sense because fertilizer used in agriculture must first reach the marine environment through leaching and transport via soil, ditches and streams. Thus, P increases with amount of fertilizer use with a time lag of one year.

### Mussel Stocks and Fertilizer Use

There was more mussel stock when fertilizer use was high in the current year (*F* = 11.37, d.f. = 1, 20, *r* ^2^ = 0.33, *P* = 0.0030, estimate (SE) = 0.005 (0.001)). No other time lag fitted the data better, nor did year enter as a significant predictor.

### Number of Eiders in Relation to Fertilizer Use and Water Temperature

**Figure 4:**
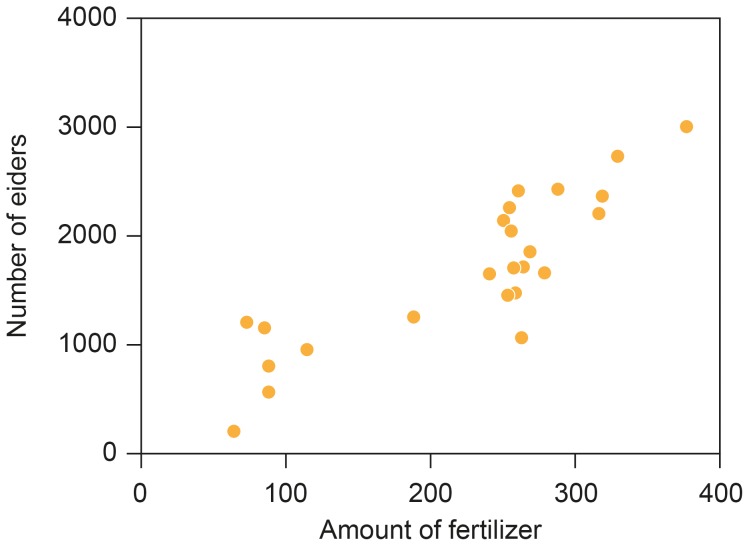
Number of breeding eiders at Christiansø in year (i) in relation to fertilizer use in Danish agriculture in the same year (year (i)) (1950 = 100).

The abundance of eiders increased with fertilizer use in year (i) (Fig. 4; *F* = 63.39, d.f. = 1, 22, *r* ^2^ = 0.73, *P*<0.0001, estimate (SE) = 6.650 (0.835)). There were no significant effects of year, fertilizer use with time lags or winter water temperature.

### Number of Eiders in Relation to Mussel Stocks

**Figure 5:**
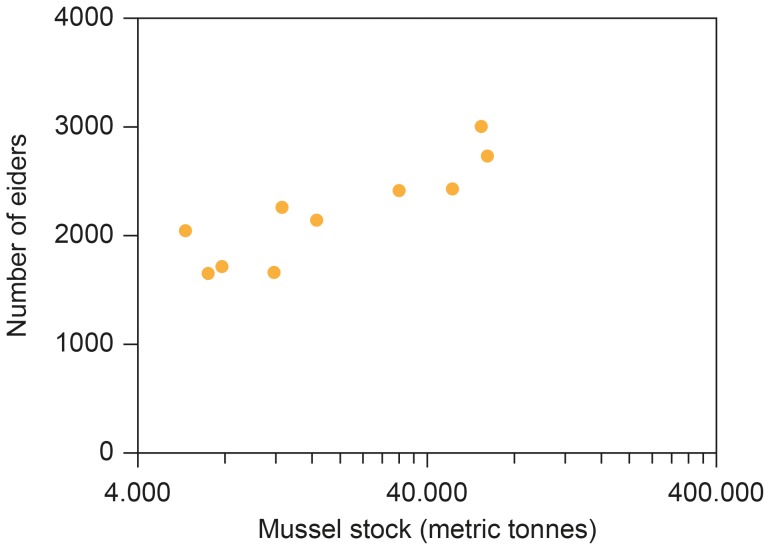
The number of breeding eiders at Christiansø in year (i) in relation to mussel stocks (metric tonnes) the same year (year (i)).

The number of breeding eiders increased with the amount of mussel stock in year (i)(Fig. 5; *F* = 26.54, d.f. = 1, 8, *r* ^2^ = 0.77, *P* = 0.0009, estimate (SE) = 1003.65 (194.80)). This model accounted for 74% of the variance in abundance of eiders. There were no significant effects of year, mussel stock with time lag or winter water temperature.

## Discussion

The number of eiders breeding at Christiansø increased from 1925 until 1992–1995, followed by a steep decrease in recent years. Estimated breeding numbers in Sweden and Finland, where the vast majority of the eider population of the Baltic/Wadden Sea flyway breeds, show a similar trend from the 1980's until the first half of the 1990's with a subsequent decline in the second part of the 1990's [12]. The two Finnish colonies Söderskär and Aasla studied during four to five decades showed peaks in breeding numbers around 1985 and 1997, respectively, both with a steep subsequent decline [19]. Countrywide monitoring of breeding eiders in Denmark at ten-year intervals showed increasing numbers up to 1990 followed by stable numbers during 2000 and 2010 [41]. Annual, systematic counts of wintering eiders in the Wadden Sea showed stable numbers until 1995 followed by declines [42]. In the Danish waters, which constitute the main wintering grounds for this population of eiders, numbers declined by 54% between 1991 and 2000 [11], [43], and for the flyway population as a whole a decline of 36% was recorded during the same period, although numbers partly recovered in recent years [12]. All these estimates from breeding and wintering grounds reveal a pronounced reduction (or stabilization in one country) in the number of eiders around 1995 with breeding numbers at Christiansø reflecting the general population trend.

However, the number of eiders on Christiansø does not reflect the total population size because non-breeding females in poor body condition probably spend the breeding season at sea, and they are thus not included in the data [12]. The number of non-breeding females is supposed to increase in periods with declining populations. This implies that the numbers of eiders in such periods probably are larger than those counted at the colony, and the population decline is smaller than reflected by the number of females in the colony. Finnish studies show that the age at first reproduction is younger when the number of eiders is increasing in a colony [39]. These two factors could have caused smaller numbers during the decline phase and larger numbers during the increase phase.

Fertilizer from agricultural areas ends up as nutrients in the marine environment where it stimulates the growth of mussel stocks. Although our data did not describe the precise time lag in the processes of transport of nutrients from farmland to the Wadden Sea, presumable due to short time series, we show a significant correlation between fertilizer use and the amount of phosphorus in the Wadden Sea and between the amount of fertilizer and mussel stocks. Furthermore, we found a strong positive correlation between mussel stocks and the number of breeding eiders at Christiansø. Finally, there was a significant correlation between mussel stocks in the Danish Wadden Sea and the number of breeding eiders at Christiansø.

Due to the close relationship found between nutrients and mussel stocks and between mussel stocks and the number of breeding eiders at Christiansø it is reasonable to assume that the reduced numbers of breeding females in the colony reflect individuals that have abandoned breeding (such females stay at sea and hence are not covered by the counts of incubating females), or individuals that have died due to reduced food availability. Females that have abandoned breeding or for other reasons have not appeared at the breeding colony have been reported from colonies in Finland [44] and on Iceland [45] in years with poor food availability. Emigration to other colonies is another explanation for the reduced number of breeders, although this explanation seems unlikely because countrywide surveys of breeding eiders in Denmark have shown stable numbers after 1995 with no increase in colonies adjacent to Christiansø [41]. In addition, adult females show an extremely high degree of philopatry close to 100% [21]. A similar degree of site fidelity by females has been reported from other colonies supporting the assumption that emigration of females does not occurred between colonies [46], [47].

Data on mussel stocks from the Danish Wadden Sea were used in our statistical models, although eiders from Christiansø also spend part of the non-breeding season in other parts of the Wadden Sea, i.e. in Germany and the Netherlands [20], [48]. However, Wadden Sea mussel stocks in Germany (both in Schleswig-Holstein and Lower Saxony) show the same pattern in abundance as in the Danish Wadden Sea with large stocks in 1998–2001 followed by low levels up to 2005, and small increases in more recent years [49]. Thus there is a common pattern in mussel stocks in major parts of the Wadden Sea, indicating that mussel stock data from the Danish part are representative of a larger region.

Eiders from Christiansø spend late winter and early spring in Kattegat, and a month before breeding they fly to the western Baltic Sea off the colony at Christiansø [20], [48]. The amount of nutrients has also decreased in the Kattegat by more than 30% since 1991–1992 [6] as in the Danish Wadden Sea. A reduction in nutrients by 50% has occurred in coastal parts of the western Baltic Sea from the late 1980's to 2006 [7]. Despite reductions in nutrients in recent years, the sea off the eider colony at Christiansø has suffered from seasonal hypoxia and oxygen depletion during summer and autumn, and this has reduced or eliminated most of the bottom fauna especially large, slow-growing species such as mussels in favour of small, rapidly reproducing organisms [7]. Studies in the Baltic Sea have shown that foraging during the pre-breeding period is important for eiders [50]. Thus hypoxia at the pre-breeding grounds and the reduction in nutrients at the wintering grounds has both reduced the abundance of mussel for eiders from Christiansø.

In conclusion, marine areas where eiders from Christiansø occur during the non-breeding season have experienced reductions in nutrient levels for more than two decades. Because low nutrient levels reduce mussel stocks the population of breeding eiders at Christiansø has declined. The population trend for the eider colony at Christiansø is representative for the general trend of the Baltic/Wadden Sea flyway population. Reductions in nutrients in the marine environment occur in several geographical areas [51] and in most parts of North-western Europe. Therefore, we hypothesize that this environmental variable account for the overall reduction in the total eider population of the Baltic and Wadden Seas. Hence, our findings may provide a general explanation for the trends in eider numbers in large parts of Western Europe.

## Supporting Information

Table S1 **Data used for the statistical analyses.** Numbers of eider at Christiansø, the amount of fertilizer used by Danish farmers (indexed 1950 = 100), water temperature (C°) during January-February (data from the Dutch Wadden Sea), mussel stocks (metric tonnes) and total nitrogen (µg/l) and total phosphorus (µg/l) in both spring and autumn in the Danish Wadden Sea.
(XLSX)

Table S2 **Summary statistics for predictor variables.**
(DOCX)

Table S3 **Correlation matrix for predictor variables.**
(DOCX)

Table S4 **Temporal trends in predictor variables.**
(DOCX)
