# Supplementary material for: Long-Term Changes in Nutrients and Mussel Stocks Are Related to Numbers of Breeding Eiders Somateria mollissima at a Large Baltic Colony
Source: PLoS One. 2014 Apr 29;9(4):e95851. doi: 10.1371/journal.pone.0095851 (PMC4004576; doi:10.1371/journal.pone.0095851)
Supplement: Table S2 — Summary statistics for predictor variables. (DOCX) [file pone.0095851.s002.docx]

**ESM Table S2. Summary statistics for predictor variables.**

| **Variable** | **Mean** | **SE** | **Minimum** | **Maximum** |
| --- | --- | --- | --- | --- |
| No. eiders | 1675 | 143 | 200 | 3000 |
| Fertilizer | 227 | 19 | 64 | 377 |
| Water temperature | 3.86 | 0.35 | -0.80 | 6.42 |
| Mussel stock | 26,897 | 7369 | 5840 | 64,680 |
| Total P in spring | 1.20 | 0.04 | 0.71 | 1.74 |
| Total N in spring | 3.21 | 0.03 | 2.88 | 3.44 |
