# Supplementary material for: Long-Term Changes in Nutrients and Mussel Stocks Are Related to Numbers of Breeding Eiders Somateria mollissima at a Large Baltic Colony
Source: PLoS One. 2014 Apr 29;9(4):e95851. doi: 10.1371/journal.pone.0095851 (PMC4004576; doi:10.1371/journal.pone.0095851)
Supplement: Table S3 — Correlation matrix for predictor variables. (DOCX) [file pone.0095851.s003.docx]

**ESM Table S3. Correlation matrix for predictor variables.**

|  | **No. eiders** | **Fertilizer** | **Water temperature** | **Precipitation** | **Mussel stocks** | **Total P in spring** |
| --- | --- | --- | --- | --- | --- | --- |
| **No. eiders** |  |  |  |  |  |  |
| **Fertilizer** | 0.87 |  |  |  |  |  |
| **Water temperature** | 0.16 | 0.12 |  |  |  |  |
| **Precipitation** | -0.58 | -0.88 | -0.01 |  |  |  |
| **Mussel stock** | 0.82 | 0.62 | 0.11 | -0.88 |  |  |
| **Total P in spring** | 0.78 | 0.82 | 0.32 | -0.65 | 0.40 |  |
| **Total N in spring** | 0.43 | -0.15 | 0.33 | 0.14 | -0.18 | 0.03 |
