# Supplementary material for: Long-Term Changes in Nutrients and Mussel Stocks Are Related to Numbers of Breeding Eiders Somateria mollissima at a Large Baltic Colony
Source: PLoS One. 2014 Apr 29;9(4):e95851. doi: 10.1371/journal.pone.0095851 (PMC4004576; doi:10.1371/journal.pone.0095851)
Supplement: Table S4 — Temporal trends in predictor variables. (DOCX) [file pone.0095851.s004.docx]

**ESM Table S4. Temporal trends in predictor variables.**

| **Variable** | ***F*** | **d.f.** | ***r*^2^** | ***P*** | **Estimate** | **SE** |
| --- | --- | --- | --- | --- | --- | --- |
| No. eiders | 19.73 | 1, 22 | 0.45 | 0.0002 | 17.52 | 3.94 |
| Fertilizer | 48.77 | 1, 22 | 0.68 | < 0.0001 | 2.74 | 0.39 |
| Water temperature | 2.09 | 1, 22 | 0.05 | 0.16 | 0.018 | 0.013 |
| Precipitation | 0.03 | 1, 19 | -0.05 | 0.86 | -0.62 | 3.38 |
| Mussel stock | 27.46 | 1, 8 | 0.77 | 0.0008 | -4642 | 886 |
| Total P in spring | 7.22 | 1, 10 | 0.36 | 0.023 | -0.68 | 0.25 |
| Total N in spring | 0.37 | 1, 19 | -0.03 | 0.55 | -0.003 | 0.005 |
